# Novel cardioprotective strategy combining three different preconditioning methods to prevent ischemia/reperfusion injury in aged hearts in an improved rabbit model

**DOI:** 10.3892/etm.2015.2680

**Published:** 2015-08-13

**Authors:** JIAN-XI YE, DAO-ZHONG CHEN

**Affiliations:** Department of Cardiovascular Surgery, Union Hospital, Fujian Medical University, Fuzhou, Fujian 350001, P.R. China

**Keywords:** aged myocardium, improved rabbit myocardial ischemia-reperfusion model, preconditioning, adenosine-enhanced ischemic preconditioning, cold crystalloid cardioplegia

## Abstract

The use of ischemic preconditioning (IPC) to protect the myocardium is usually not effective in elderly patients. The aim of the present study was to design new methods to achieve enhanced myocardial protection, based on the differential role of endogenous adenosine (ADO) and ADO receptors (ARs) in the effects of IPC on young and old animals. An improved New Zealand white rabbit model of ischemia/reperfusion was established based on the Langendorff model. Adult or elderly rabbit hearts, with or without exposure to IPC, were used in order to assess the roles of ADO and ARs in the different effects of IPC. Different protective methods were designed based on a combination of endogenous and exogenous interventions. Cardiac function, as well as biochemical, histopathological and apoptotic indices, were measured in the different intervention groups. The improved Langendorff model was stable, reliable and suitable for the undertaking of the experiments. The ADO levels in the aged rabbit hearts pre- and post-IPC were lower than those in the adult hearts, indicating that ADO levels may be an endogenous factor influencing IPC. A new protection strategy combining ADO-enhanced IPC, A_1_AR agonist 2-chloro-N(6)-cyclopentyladenosine preconditioning and cold crystalloid cardioplegia had a significant protective effect in aged hearts. The results of the present study suggested that endogenous ADO enhancement, A_1_AR agonist preconditioning and exogenous treatment yield an additive effect in aged rabbit hearts. The simultaneous application of these three types of intervention provided the most effective myocardial protection in the improved aged rabbit heart model.

## Introduction

Cardiovascular diseases are common in the older population, with numerous patients undergoing coronary artery bypass grafting or cardiac valve replacement (1). Despite improvements in surgical care, ischemia and reperfusion (IR) during cardiac surgery remains a major cause of myocardial injury (2,3). Reperfusion exacerbates the ischemia-induced inflammatory response (4), thus rendering the protection of the myocardium from IR injury extremely important.

Ischemic preconditioning (IPC) remains one of the most effective strategies used to protect the myocardium (5). It uses repeated short periods of ischemia to activate the myocardium in order for it to become more resistant to subsequent ischemic insults (6). IPC consists of an early phase, which starts a few minutes after a short ischemic stimulation and lasts for 2–4 h, and a late phase, which is usually observed 24 h later (7).

Cellular kinases, such as protein kinase C (PKC), play an important role in the cardioprotective effects of IPC (8). The upregulation of protective genes is necessary for the development of the late phase of IPC (9,10). Adenosine (ADO), which is released by ischemic cells, binds to cardiac receptors (11,12), triggering signal transductions that ultimately activate PKC and result in cardioprotection (13,14). On that basis, a novel concept termed adenosine-enhanced ischemic preconditioning (APC) was proposed (5) and has been shown to enhance the cardioprotection achieved by IPC (15); however, little has been reported regarding the use of APC for myocardial protection in the elderly.

A more comprehensive understanding of the IPC mechanisms and its protective phases may enable more effective interventions in elderly patients. A reduction in the protective effects of IPC has been observed in the elderly, possibly due to a decreased capacity of their body for ADO synthesis (11,12), impaired PKC translocation in response to IPC (13), blunted sensitivity to p38 mitogen-activated protein kinase and heat shock protein 27 and/or enhanced dephosphorylation of protective proteins (15). In addition, it has been shown that a loss of ADO-induced cardioprotection is observed in aged hearts due to an age-related decline in the functionality of ventricular ADO receptors (ARs) (16). Furthermore, a previous study suggested that the loss of ADO-induced cardioprotection is not due to a decreased expression of ARs, but rather to impaired downstream signaling elements (17).

The Langendorff model for investigating isolated hearts was introduced in 1895 (18). This model is reproducible and allows investigators to study the heart without neurohumoral interference, under controlled conditions and with direct access to the areas of interest (19,20); however, this model has been shown to have disadvantages, such as high coronary flow rate, limited supply of high-energy phosphate, a reduced work output and oxygen requirement, and the risk of incorrect and poor experimental procedure (20,21). An improved version of this model is required for its use in the modeling of IPC and APC, particularly during the extraction of the heart, in order to avoid sudden cardiac arrest and minimize warm ischemic time. Despite its limitations, however, the Langendorff model has been proven useful in the study of IR (22).

The present study was conducted with the purpose of establishing a reliable model of aged rabbit hearts based on the Langendorff method. Adult and elderly rabbits were subjected to IPC. ADO levels and AR expression were detected to investigate the factors that weaken the myocardial protective effect of IPC in elderly hearts. Different protective approaches, including ADO enhancement, AR agonist administration and cold crystalloid cardioplegia, were subsequently tested alone and in combination with one another. The results of the present study may provide insights towards the improvement of cardioprotection in the elderly.

## Materials and methods

### 

#### Animals and ethics

Elderly New Zealand white rabbits (n=64), aged 137±1 weeks and weighing 3.5±0.2 kg, and adult rabbits (n=16), aged 28±1 weeks and weighing 2.8±0.1 kg, were obtained from the SLAC Laboratory Animal Center Co., Ltd. (Shanghai, China). All rabbits were housed separately, fed with a standard laboratory diet and provided with water.

All experiments were approved by the Animal Care and Use Committee of Fujian Medical University (Fuzhou, China). The study was performed in accordance with the Principles of Laboratory Animal Care, proposed by the National Society for Medical Research, and the Guide for the Care and Use of Laboratory Animals (National Institutes of Health Publication no. 5377-3, 1996). Surgeries were performed under anesthesia and analgesic agents were applied immediately at the end of surgery. All surgeries and associated tests were performed in a blinded manner.

#### Establishment of the isolated heart model and sample collection

Animals were anesthetized with pentobarbital sodium (30 mg/kg) and heparinized (200 U/Kg) via the marginal ear vein. During the isolation, hearts were perfused at 4°C with cardioplegic St. Thomas II (ST) solution (15 ml/kg; Nanjing Jiancheng Bioengineering Institute, Nanjing, China) for 2 min. Following aortic cannulation and cardiac arrest, the hearts were immersed in ST solution at 4°C. The hearts were subjected to Langendorff perfusion at a constant pressure of 60 mmHg (80 cm H_2_O) at 37°C. Cardiac function indices, including systemic arterial pressure (SAP), left ventricular systolic pressure (LVSP), changes in pressure over time (+dp/dt max and -dp/dt max), heart rate (HR) and coronary sinus flow (CSF), were monitored using an ALC_B10_-MPA myocardial function analyzing system (Alcott Biotech, Shanghai, China), and well-preserved isolated hearts were then assigned to groups according to the IR process and the different protective strategies. Aortic and CSF data were collected for analyses, and left ventricular apical myocardial tissue was extracted and frozen for further analysis.

#### IPC grouping

Sixteen adult and 16 elderly New Zealand white rabbits were divided into four groups: Adult heart control, adult heart + IPC, aged heart control and aged heart + IPC (n=8 per group).

IPC was performed in hearts from rabbits of the IPC groups. Hearts of the anesthetized aged rabbits were rapidly excised, transferred to a Langendorff apparatus and perfused via aortic cannula with an oxygenated Krebs-Henseleit buffer (Nanjing Jiancheng Bioengineering Institute) containing 118.3 mmol/l NaCl, 2.7 mmol/l KCl, 1.0 mmol/l MgSO_4_, 1.4 mmol/l KH_2_PO_4_, 29.0 mmol/l NaHCO_3_, 3.4 mmol/l CaCl_2_ and 10 mmol/l glucose, with 70 mU/l insulin and 0.4% bovine serum albumin, at 37°C. Flow was adjusted to achieve a retrograde perfusion pressure of 60 mmHg. All hearts were initially perfused with the oxygenated buffer for a 15-min stabilization period. A subset of the hearts (adult heart + IPC and aged heart + IPC) was subjected to a preconditioning protocol consisting of one cycle of global no-flow ischemia (5 min) followed by normal perfusion (5 min), prior to 120 min of global ischemia, as previously described (13). The remaining hearts were not preconditioned but were instead perfused with a normoxic buffer (Nanjing Jiancheng Bioengineering Institute) for 10 min, prior to being subjected to 120 min of global ischemia. Isolated myocardial tissue from the left ventricular apex was immediately collected for analysis.

#### ADO production

Myocardial tissue of the left ventricular apex was extracted from all hearts and immediately preserved in liquid nitrogen. ADO production in the myocardium was detected using a high-performance liquid chromatography system (Beckman System Gold; Beckman Coulter, Beijing, China). ADO standard product was purchased from Sigma-Aldrich (St. Louis, MO, USA) (23).

#### Reverse transcription-quantitative polymerase chain reaction (RT-qPCR)

Total RNA was extracted from the myocardium using the RNAiso Plus reagent (Takara Bio Inc., Shiga, Japan), and cDNA was prepared using the Primescript RT Reagent (Takara). An ABI StepOnePlus™ Real-Time-PCR System (Applied Biosystems; Life Technologies, Carlsbad, CA, USA) was used with the SYBR® Green Master Mix (Applied Biosystems), and primers were obtained from the Beijing Genomics Institute (Shenzhen, China) for the measurement of the expression of ARs (A_1_AR, A_2A_AR and A_3_AR), B-cell lymphoma-2 (Bcl-2) and intercellular adhesion molecule-1 (ICAM-1) (Table I). PCR cycling conditions were as follows: 2 min pre-denaturation at 95°C, 40 cycles of 10 sec denaturation at 95°C, 10 sec annealing at 61°C and a 40-sec extension step at 72°C. GAPDH was used as a reference to obtain the relative fold change for targets using the comparative Ct method.

#### Cardioprotection treatments

**Figure 1.**
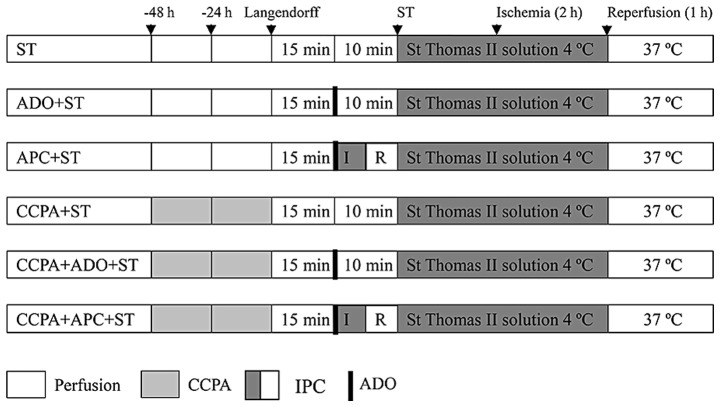
Different cardioprotection strategies: ST, ADO + ST, APC + ST, CCPA + ST, CCPA + ADO + ST and CCPA + APC + ST. CCPA treatment comprised ear vein injections of CCPA (100 µg/kg) twice, at 48 and 24 h before surgery. IPC comprised 5 min of ischemia and 5 min of reperfusion at 37°C, followed by preservation with ST solution at 4°C. ADO treatment comprised 10 ml ADO/KOH (1 mmol/l). In the ST group, Langendorff perfusion was performed for 10 min at 37°C, followed by preservation with ST solution at 4°C. ST, St. Thomas II; ADO, adenosine; CCPA, 2-chloro-N(6)-cyclopentyladenosine; APC, adenosine-enhanced ischemic preconditioning; IPC, ischemic preconditioning; IR, ischemia/reperfusion.

Cardioprotection strategies are summarized in Fig. 1. Forty-eight elderly New Zealand white rabbits were divided into six groups (n=8 per group). Following exposure to different interventions, the isolated hearts were perfused for 15 min to establish stable hemodynamics. The following six different interventions were undertaken prior to cardiac arrest for 120 min at 15°C: i) ST group, hearts were perfused with Langendorff solution for 10 min at 37°C, followed by protection with ST solution (20 ml/kg) at 4°C. The hearts were stored in the ST solution at 4°C for 120 min; ii) ADO + ST group, hearts were subjected to a 10-ml bolus injection of ADO/KOH (1 mmol/l), followed by Langendorff perfusion for 10 min at 37°C and protection with ST solution (20 ml/kg) at 4°C. The hearts were stored in the ST solution at 4°C for 120 min; iii) APC + ST group, hearts were subjected to a 10-ml bolus injection of ADO/KOH (1 mmol/l), followed by 5 min of ischemia and 5 min of reperfusion at 37°C, then protection with ST solution (20 ml/kg) at 4°C. The hearts were stored in the ST solution at 4°C for 120 min; iv) A1AR agonist 2-chloro-N(6)-cyclopentyladenosine (CCPA) + ST group, rabbits received ear vein injections of CCPA (100 µg/kg) at 48 and 24 h prior to surgery. The hearts were subjected to Langendorff perfusion for 10 min at 37°C followed by protection with ST solution (20 ml/kg) at 4°C. The hearts were stored in ST solution at 4°C for 120 min; v) CCPA + ADO + ST group, rabbits received ear vein injections of CCPA (100 µg/kg) at 48 and 24 h prior to surgery. Hearts were subjected to a 10-ml bolus injection of ADO/KOH (1 mmol/l), followed by Langendorff perfusion for 10 min at 37°C and protection with ST solution (20 ml/kg) at 4°C. The hearts were stored in ST solution at 4°C for 120 min; vi) CCPA + APC + ST group, rabbits received ear vein injection of CCPA (100 µg/kg) at 48 and 24 h prior to surgery. The hearts were subjected to a 10-ml bolus injection of ADO/KOH (1 mmol/l), followed by 5 min ischemia and 5 min reperfusion at 37°C, followed by protection with ST solution (20 ml/kg) at 4°C. The hearts were stored in ST solution at 4°C for 120 min.

#### Cardiac function indices

Cardiac function indices, including SAP, LVSP, +dp/dt, -dp/dt, HR and CSF, were checked before treatment and 30/60 min post-treatment, using the ALC_B10_-MPA Cardiac Function Analysis System (Alcott Biotech), according to the manufacturer's instructions. Post-treatment hemodynamic parameters were measured by catheterization (SRP-320/PVAN 3.2, Millar Instruments, Inc., Houston, TX, USA; Chart 5 software, ADInstruments, Dunedin, New Zealand), as previously described (24).

#### Myocardial enzyme leakage

Coronary venous outflow (2 ml) was collected prior to cardiac arrest and 20 min after reperfusion. The levels of myocardial enzyme leakage in the preserved liquid were measured in duplicate using a creatine kinase (CK) and lactate dehydrogenase (LDH) ELISA testing kit (Nanjing Jiancheng Bioengineering Institute), according to the manufacturer's instructions.

#### Endothelial function testing

Coronary venous outflow (2 ml) was collected prior to cardiac arrest and 30 min after reperfusion. Levels of NO and endothelin (ET) were detected using NO and ET testing kits (Nanjing Jiancheng Bioengineering Institute), according to the manufacturer's instructions.

#### Malondialdehyde (MDA) and superoxide dismutase (SOD) detection

Coronary venous outflow (2 ml) was collected prior to cardiac arrest and 60 min after reperfusion. MDA and SOD levels were measured using MDA and SOD testing kits (Nanjing Jiancheng Bioengineering Institute), according to the manufacturer's instructions.

#### Adenosine triphosphate (ATP) in myocardial tissue

Myocardial tissues of the left ventricular apex were collected 60 min after reperfusion. ATP levels were detected using an ATP testing kit (Beyotime Institute of Biotechnology, Shanghai, China).

#### Histopathology

Myocardial tissues of the left ventricular apex were collected and fixed in 4% formalin, dehydrated and embedded in paraffin. The samples were then sectioned and stained using standard methods. Samples were observed by microscopy (Olympus Corp., Tokyo, Japan) at x100 and x400 magnifications.

#### Electron microscopy

Myocardial tissues of the left ventricular apex were collected and prepared for electron microscopy, as previously described (25).

#### Apoptosis analysis

The apoptotic index of the myocardium was assessed by the terminal deoxynucleotidyl transferase-mediated dUTP nick end labeling (TUNEL) method using a TUNEL staining kit (Fuzhou Maxim Biotech, Inc., Fuzhou China), according to the manufacturer's instructions. Samples were examined under microscopy and stained cells were counted in six random visual fields.

#### Statistical analysis

Statistical analysis was performed using SPSS 17.1 software (SPSS Inc., Chicago, IL, USA). Data are presented as the mean ± standard error. Two-way repeated-measures analysis of variance was performed to analyze the differences between the groups at different time-points. P<0.05 was considered to indicate a statistically significant difference.

## Results

### 

#### Establishment of the improved Langendorff-perfused isolated heart and myocardial IR injury models

Isolated heart models were successfully established with almost no warm ischemic time in New Zealand white rabbits. All animals had a strong heartbeat during surgery and no cardiac arrest occurred. The duration between surgery and Langendorff perfusion ranged from 9 to 10 min, including 4 min of cold ischemia. Cardiac function was preserved in all isolated hearts, as determined by the following indices: SAP, 83.98±2.18 mmHg; LVSP, 90.01±2.34 mmHg; +dp/dt, 1,840.15±52.74 mmHg/sec; -dp/dt, 1,438.75±62.17 mmHg/sec; HR, 193.94±6.97 bpm and CSF, 60.96±3.09 ml/min. These models were suitable to fulfill the demands of the following experiments.

#### Endogenous ADO levels and AR expression are involved in the protective effect of IPC

The baseline levels of endogenous ADO were found to be significantly lower in the aged groups than those in the adult groups (P<0.05, Table II). The increase in ADO in the aged + IPC group was significantly lower than that in the adult + IPC group (P<0.05). These findings indicated that endogenous ADO was associated with the protective effects of IPC in aged rabbit hearts.

As shown in Table II, the baseline levels of A_1_AR, A_2A_AR and A_3_AR were decreased in the aged control group compared with those in the adult control group (P<0.05); however, following IPC, the AR expression was regulated in different manners. The mRNA levels of A_1_AR and A_3_AR increased more significantly in the aged + IPC group than those in the adult + IPC group (P<0.05). Following IPC, a 4.36-fold increase was observed in the A_1_AR and a 1.87-fold increase in the A_3_AR mRNA levels in the aged heart tissue, compared with the 1.51-fold increase in the A_1_AR mRNA levels and the 1.68-fold increase in the A_3_AR mRNA levels in the adult heart tissue. The mRNA levels of A_2A_AR were significantly lower in the aged + IPC group than those in the adult + IPC group (P<0.05).

#### Cardiac function is preserved using a combination of CCPA, APC and cold crystalloid cardioplegic solution in aged rabbit hearts

**Figure 2.**
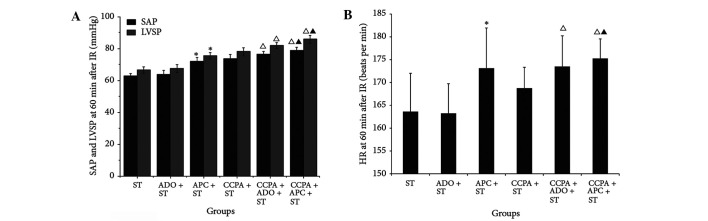
Cardiac and endothelial functions and oxidation of elderly rabbit hearts in the ST, ADO + ST, APC + ST, CCPA + ST, CCPA + ADO + ST and CCPA + APC + ST groups. (A) SAP and LVSP and (B) HR were detected 60 min after IR. *P<0.05 vs. ST group; ^Δ^P<0.05 vs. APC + ST group; ^▲^P<0.05 vs. CCPA + ST group. ST, St. Thomas II solution; ADO, adenosine; APC, adenosine-enhanced ischemic preconditioning; CCPA, 2-chloro-N(6)-cyclopentyladenosine; SAP, systemic arterial pressure; LVSP, left ventricular systolic pressure; IR, ischemia/reperfusion; ATP, adenosine triphosphate; HR, heart rate.

The results in Table III show that the baseline values of SAP, LVSP, +dp/dt and -dp/dt were similar in the CCPA + ST, CCPA + ADO + ST and CCPA + APC + ST groups (P>0.05). Sixty minutes after IR, the values of SAP, LVSP, +dp/dt and -dp/dt were improved in the combination groups, with the most marked change observed in the CCPA + APC + ST group (P<0.05, Fig. 2A). It was also found that, 60 min after IR, the HR and CSF were improved in the combination groups, particularly in the CCPA + APC + ST group, where the improvement was more marked than that in the other groups (P<0.05, Fig. 2B and Table III).

**Figure 3.**
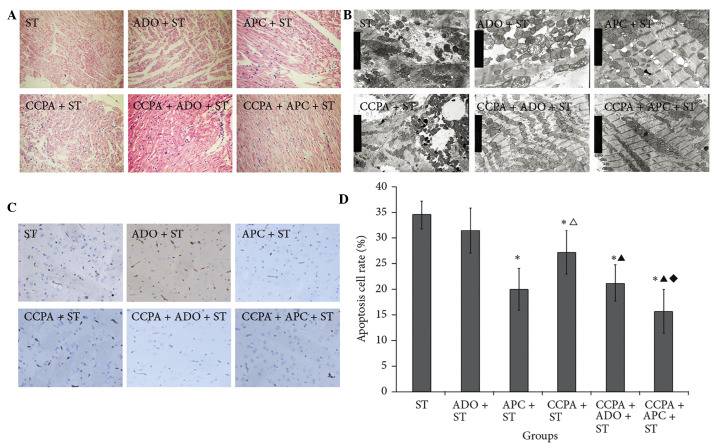
(A) Hematoxylin and eosin staining (magnification, x400), (B) ultrastructure (magnification, x8,000) and (C) terminal deoxynucleotidyl transferase-mediated dUTP nick end labeling staining (magnification, x400) of elderly rabbit myocardial fibers in the ST, ADO + ST, APC + ST, CCPA + ST, CCPA + ADO + ST and CCPA + APC + ST groups. (D) Quantitative data of (C). *P<0.05 vs. ST group; ^Δ^P<0.05 vs. APC + ST group; ^▲^P<0.05 vs. CCPA + ST group; ^♦^P<0.05 vs. CCPA + ADO + ST group. ST, St. Thomas II; ADO, adenosine; APC, adenosine-enhanced ischemic preconditioning; CCPA, 2-chloro-N(6)-cyclopentyladenosine.

Myocardial samples from the CCPA groups showed an indefinable distribution of hematoxylin and eosin (H&E) stain. Myocardial fibers were well preserved, although moderate regional swelling was observed in the intercellular matrix. In the CCPA + APC + ST group, the fibers were arranged in neat rows, the cell size was uniform and the H&E staining was well distributed. There was no obvious cellular swelling (Fig. 3A).

Transmission electron microscopy revealed that, following IR, myocardial fibers from the different groups varied in size and shape and some exhibited mitochondrial hyperplasia. Both prior to and following IR, the myocardial fibers in the ST group were abnormal, with clear swelling observed in the cytoplasm and mitochondria. By contrast, following IR, myocardial fibers from the CCPA + APC + ST group showed a clear, normal structure and contained rows of mitochondria without evidence of swelling (Fig. 3B).

#### CCPA + APC+ ST treatment prevents myocardial apoptosis by reducing enzyme leakage, increasing levels of ATP, Bcl-2 and ICAM-1, improving endothelial function and protecting the mitochondrial function of the myocardium

As shown in Fig. 3C, significantly fewer apoptotic cells were found in the treatment groups, with the exception of the ADO + ST group, compared with the ST group (P<0.05). No significant difference in the apoptotic index was observed between the APC + ST and CCPA + ADO + ST groups; however, the apoptotic index was considerably lower in the CCPA + APC + ST group compared with the other groups (P<0.05), suggesting that both CCPA and APC protected myocardial cells from IR-induced injury, and therefore supporting the combined use of these two agents.

The levels of Bcl-2, ICAM-1, CK and LDH, indicators of cardiac injury or inflammation, were subsequently investigated. As shown in Table IV, the expression of Bcl-2, which is a factor that protects cells from apoptosis, was increased in all treatment groups, with the exception of the ADO + ST group, compared with that in the ST group (P<0.05). The levels of ICAM-1, a factor associated with the induction of inflammation, were also decreased in the treatment groups, with the exception of the ADO + ST group, compared with the ST group (P<0.05). The expression of Bcl-2 in the CCPA + APC + ST group was considerably higher than that in any of the other groups (P<0.05) and the ICAM-1 levels were significantly lower than those in any of the other groups (P<0.05).

**Figure 4.**
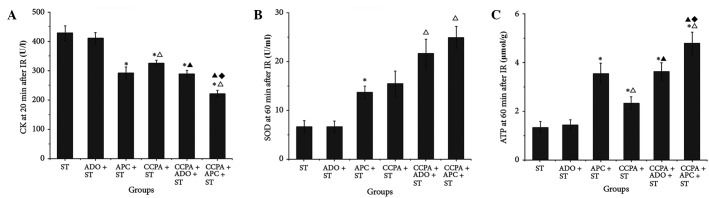
Cardiac and endothelial functions and oxidation of elderly rabbit hearts in the ST, ADO + ST, APC + ST, CCPA + ST, CCPA + ADO + ST and CCPA + APC + ST groups. (A) CK was detected 20 min after IR; (B) SOD and (C) ATP were detected 60 min after IR. *P<0.05 vs. ST group; ^Δ^P<0.05 vs. APC + ST group; ^▲^P<0.05 vs. CCPA + ST group; ^♦^P<0.05 vs. CCPA + ADO + ST group. ST, St. Thomas II solution; ADO, adenosine; APC, adenosine-enhanced ischemic preconditioning; CCPA, 2-chloro-N(6)-cyclopentyladenosine; IR, ischemia/reperfusion; CK, creatine kinase; SOD, superoxide dismitase; ATP, adenosine triphosphate.

CK and LDH are important indicators of myocardial injury. In order to assess the extent of IR injury, coronary venous outflow was collected prior to and 20 min after IR. As shown in Table III, no differences were identified among the groups in the levels of CK and LDH prior to IR (P>0.05); however, 20 min after IR, the CK and LDH leakage was significantly increased in each of the studied groups (P<0.05), but the lowest value was observed in the CCPA + APC + ST group (P<0.05, Fig. 4A and Table III).

NO and ET levels are indicators of endothelial function. As shown in Table III, prior to ischemia, NO levels were higher in the CCPA-treated groups than those in the other groups. Following IR, NO production was significantly reduced, but the best recovery was observed in the CCPA + APC + ST group (P<0.05). Changes in ET levels showed an inverse association; CCPA + APC + ST inhibited the abnormal increase in ET production following IR.

MDA, SOD and ATP, as indicators of oxidative status, were measured prior to and following IR. The results shown in Table III revealed that the mitochondrial function of the myocardium was the most effectively protected, and the levels of reactive oxygen species (ROS) were the lowest, in the CCPA + APC + ST group (P<0.05, Fig. 4B and C).

## Discussion

It has been previously shown that IPC renders the myocardium resistant to subsequent ischemic insult (14). Although IPC has a beneficial effect on IR (15), the most suitable approaches for specific populations remain controversial. In the present study, a modified model of aged and adult rabbit hearts was established based on the Langendorff model, in order to explore the myocardial protective role of ADO and ARs. The protective effect of APC in combination with pharmacological preconditioning and cold crystalloid cardioplegia solution was also studied. The results showed that a combination approach was more effective in preserving cardiac function, preventing myocardial apoptosis and reducing ROS levels in aged hearts compared with single intervention approaches.

The isolated retrograde-perfused Langendorff heart has previously been shown to be invaluable in studying the pharmacological effects of different agents on myocardial function and disease states such as IR injury (26). To successfully establish an isolated heart model, it is necessary to avoid sudden cardiopulmonary arrest, provide adequate time for myocardial preservation and cold ischemia and ensure that an accurate surgical technique is being used. These aims were achieved in the present study and an improved isolated rabbit heart model with almost no warm ischemic time and an optimal protection of cardiac function during cold ischemia was established. This improved model could provide better insight into the study of IR injury and its prevention. Traditional isolated heart models have several limitations, such as a high incidence of pneumothorax, damage to the left atrium and absence of a protective strategy during myocardial preservation and cold ischemia, which render the isolated heart models relatively unstable with high failure rates. In the present study, several improvements were applied to the preparation of the isolated heart model. First, pentobarbital sodium was administered following the administration of heparin in an attempt to avoid sudden cardiopulmonary arrest. A medical ventilator was used to limit the impact of pneumothorax on respiratory and circulatory function. In order to ensure zero warm ischemic time, exogenous protection using ST solution maintained at 4°C was applied during the surgical and isolation processes. In addition, the KOH and ST solutions were maintained within standard ranges and at moderate constant pressure to avoid heart failure. Other improvements included the full drainage of the left and right ventricles prior to perfusion, in order to prevent heart injury due to filling, and the non-conventional placement path of the perfusion and piezometric tubes. These improvements to the standard technique provided a stable, practical and easily reproducible model, which allowed the measurement of cardiac function indices, such as SAP, LVSP, +dp/dt, -dp/dt, HR and CSF. These parameters were well preserved in all hearts included in the present study and allowed a comprehensive assessment of the subsequent IR, IPC and APC processes.

The AR family includes four subtypes, among which A_1_AR, A_2A_AR and A_3_AR are closely associated with cardiac function (27,28). Studies using IR models have shown that A_1_AR activation significantly decreases myocardial infarct size and enhances post-ischemic functional recovery (5,15). In the present study, differential regulation of A_1_AR, A_2A_AR and A_3_AR expression was observed in adult and elderly isolated rabbit heart models prior to and following IPC. These data revealed that factors other than the expression of ARs, such as downstream effectors of ARs, may have important effects on heart protection; these require further study.

CCPA is a highly selective A_1_AR agonist that has been shown to induce the late-phase protective effect of IPC (29). APC extends the cardioprotection of IPC by interacting with ARs, primarily during IR (30). Other studies advocate the use of pharmacological preconditioning to reduce IR injury and increase the time available for effective reperfusion (7,9,31,32). In the present study, following the administration of a high dose of CCPA in combination with APC, fully activated A_1_AR significantly improved the protective effect of APC in aged myocardial tissue. ST solution is a classical exogenous method used to protect the myocardium. A further combination of APC with CCPA and ST solution had the highest performance in cardioprotection compared with all other tested approaches, indicating that the early- and late-phase endogenous protection combined with exogenous protection could be used simultaneously and result in an additive effect in aged hearts.

The combination treatment also enabled endothelial cells to produce more NO, thereby reducing harmful ET, preserving the function of the myocardium and stabilizing HR. In addition, the apoptotic index, the function and number of mitochondria, the ATP levels and the expression of Bcl-2, ICAM-1, CK and LDH were all maintained in a more favorable range in the CCPA + APC + ST group compared with the ranges observed in the other groups.

The present study was limited, as it was mostly observational, and further studies are required to assess the exact molecular mechanisms involved in IPC and APC, as well as the exact differences between adult and aged hearts.

In conclusion, the findings of the present study suggest that the simultaneous combination of endogenous and exogenous protective methods could prove beneficial to the protection of aged myocardium from IR injury; therefore, the present study could provide a basis for clinical application in elderly patients during surgery.

## Figures and Tables

**Table I.** Reverse transcription-quantitative polymerase chain reaction primer sequences.

Genes	Sequences	Length (bp)
GADPH	Forward: 5′-ACCACAGTCCATGCCATCAC-3′	440
	Reverse: 5′-TCCACCACCCTGTTGCTGTA-3′	
A_1_AR	Forward: 5′-GCTACCACCCCTTGGACATAAC-3′	190
	Reverse: 5′-TGGGCACATCAGCAGACAGG-3′	
A_2A_AR	Forward: 5′-TTCGCCATCACCATCAGCAC-3′	177
	Reverse: 5′-CCTCATACCCGTCACCAAGC-3′	
A_3_AR	Reverse: 5′-AGAACGGTTACCACTCAAAGAAG-3′	166
	Reverse: 5′-AACTGACCACGGAACGGAAG-3′	
Bcl-2	Forward: 5′-AGTGGGATACTGGAGATGAAGAC-3′	234
	Reverse: 5′-GACGGTAGCGACGAGAGAAG-3′	
ICAM-l	Forward: 5′-TGAGAAATTGGCTCCGTGGTC-3′	103
	Reverse: 5′-CCGTGGGAATGAGACACTGAG-3′	

Bcl-2, B-cell lymphoma-2; ICAM-l, intercellular adhesion molecule; AR, adenosine receptor.

**Table II.** ADO levels and relative expression of A_1_AR, A_2_AR and A_3_AR mRNA.

Groups	ADO (mg/g)	Fold change after IPC	A_1_AR/GADPH	Fold change after IPC	A_2A_AR/GADPH	Fold change after IPC	A_3_AR/GADPH	Fold change after IPC
Adult	0.29±0.03		1.00		1.00		1.00	
Adult + IPC	1.23±0.18	4.28	1.51±0.25^a^	1.51	0.51±0.06^a^	0.51	1.68±0.20^a^	1.68
Aged	0.19±0.03^a^		0.55±0.04^a^		0.62±0.11^a^		0.39±0.07^a^	
Aged + IPC	0.63±0.04^a^	3.33	2.40±0.34^a–c^	4.36	0.23±0.03^a–c^	0.37	0.73±0.21^a–c^	1.87

Data are presented as the mean ± standard error.

a P<0.05 compared with the adult heart group

b P<0.05 compared with the aged heart group

c P<0.05 compared with the adult heart + IPC group. ADO, adenosine; IPC, ischemic preconditioning; AR, adenosine receptor.

**Table III.** Values of indicators of cardiac and endothelial function, myocardial enzyme leakage and levels of reactive oxygen species, SOD and ATP.

Indicators	Time-points	ST	ADO + ST	APC + ST	CCPA + ST	CCPA + ADO + ST	CCPA + APC + ST
SAP (mmHg)	Prior to ischemia	80.61±1.79	81.68±3.14	81.60±1.96	86.53±2.19^a^	86.70±2.12^a^	86.77±1.88^a^
	30 min after IR	68.32±1.97	69.08±3.93	75.49±2.92^a^	76.84±2.01	79.92±2.22^b^	82.22±1.83^b,c^
	60 min after IR	63.00±1.33	63.53±2.97	71.91±2.54^a^	73.76±2.50	76.37±2.06^b^	78.91±1.94^b,c^
LVSP (mmHg)	Prior to ischemia	85.01±2.13	85.42±2.70	86.40±2.44	94.45±2.91^a^	93.77±1.68^a^	95.01±2.15^a^
	30 min after IR	71.06±2.16	71.62±2.09	78.31±2.49^a^	82.33±1.94	84.95±2.21^b^	89.83±1.88^b,c^
	60 min after IR	66.54±2.02	67.48±2.40	75.56±1.85^a^	78.05±2.54	81.91±1.95^b^	85.74±2.47^b,c^
+dp/dt (mmHg/sec)	Prior to ischemia	1,728.26±58.24	1,698.35±65.59	1,716.42±38.61	1,955.75±62.37^a^	1,966.48±40.21^a^	1,975.63±51.42^a^
	30 min after IR	1,341.48±87.34	1,356.65±76.28	1,537.23±96.54^a^	1,662.65±92.28	1,747.61±83.61^b^	1,826.76±72.78^b,c^
	60 min after IR	1,292.22±112.31	1,285.46±110.21	1,502.21±82.25^a^	1,577.12±72.62	1,706.71±90.27^b^	1,782.81±88.35^b^
−dp/dt (mmHg/sec)	Prior to ischemia	1,321.28±65.23	1,287.86±80.16	1,305.39±58.24	1,562.54±73.26^a^	1,573.61±52.38^a^	1,581.82±43.72^a^
	30 min after IR	1,030.73±85.38	1,004.56±69.52	1,156.84±79.63^a^	1,324.88±82.35	1,385.25±72.19^b^	1,457.01±65.84^b,c^
	60 min after IR	972.73±92.46	963.24±88.47	1,130.08±75.48^a^	1,252.53±69.38	1,350.47±57.63^b^	1,426.01±80.86^b,c^
HR (beats/min)	Prior to ischemia	191.88±10.01	194.25±8.33	193.00±9.06	195.25±4.95	194.50±5.68	194.75±3.81
	30 min after IR	179.00±8.96	180.25±6.61	182.75±8.38	183.75±4.74	182.25±6.92	183.13±4.91
	60 min after IR	163.63±8.38	163.25±6.48	173.13±8.82^a^	168.75±4.59	173.50±6.72^a^	175.25±4.30^a^
CSF (ml/min)	Prior to ischemia	54.91±3.08	55.40±2.28	55.15±2.61	66.13±3.12^a^	66.66±3.62^a^	67.48±3.80^a^
	30 min after IR	41.49±2.05	45.81±2.41^a^	48.84±2.73^a^	54.21±2.80	58.70±3.68^b^	61.80±3.37^b,c^
	60 min after IR	32.53±1.89	39.36±1.94^a^	42.62±2.22^a^	47.17±2.67	50.95±3.06^b^	55.82±3.21^b,c^
CK (U/l)	Prior to ischemia	64.88±6.87	72.07±8.10	67.91±4.60	66.11±3.58	66.68±4.12	67.54±4.17
	20 min after IR	428.88±24.54	411.29±19.69	293.20±20.77^a^	327.54±8.98^a,b^	289.46±11.47^a,c^	223.69±10.10^a–d^
LDH (U/l)	Prior to ischemia	22.19±3.16	22.78±3.58	22.82±3.24	23.05±3.28	23.21±2.97	22.47±3.36
	20 min after IR	90.53±4.65	89.57±3.77	74.74±4.13^a^	80.21±4.95^a,b^	75.74±3.99^a,c^	60.20±4.04^a–d^
NO (µmol/ml)	Prior to ischemia	76.91±3.35	80.69±4.40	78.44±3.57	92.37±2.04^a^	94.34±2.50^a^	95.06±1.77^a^
	30 min after IR	29.90±1.13	36.04±1.97^a^	38.24±2.21^a^	41.32±2.79	45.54±3.21^b^	54.27±3.67^b,c^
ET (ng/ml)	Prior to ischemia	31.13±2.76	30.04±2.07	30.16±2.74	24.81±4.28^a^	23.93±4.62^a^	25.23±5.26^a^
	30 min after IR	76.56±6.95	53.99±3.13^a^	48.42±4.63^a^	52.09±10.8	40.80±9.07^b^	32.83±5.77^b,c^
MDA (nmol/ml)	Prior to ischemia	8.57±1.90	10.03±1.56	8.34±2.26	5.19±1.43^a^	4.81±1.02^a^	4.49±1.20^a^
	60 min after IR	22.48±4.83	21.08±2.21	16.62±1.97^a^	11.57±3.14	10.13±2.36	6.98±1.61^b,c^
SOD (U/ml)	Prior to ischemia	17.58±2.03	16.46±2.79	17.35±1.93	27.46±1.92^a^	27.99±2.34^a^	28.52±2.32^a^
	60 min after IR	6.65±1.25	6.65±1.13	13.72±1.29^a^	15.46±2.60	21.70±2.92^b^	24.98±2.24^b^
ATP (µmol/g)	60 min after IR	1.34±0.21	1.44±0.20	3.55±0.40^a^	2.33±0.26^a,b^	3.62±0.36^a,c^	4.77±0.47^a^

Data are presented as the mean ± standard error.

a P<0.05 vs. the ST group

b P<0.05 vs. the APC + ST group

c P<0.05 vs. the CCPA + ST group

d P<0.05 vs. the CCPA + ADO + ST group. ST, St. Thomas II solution; ADO, adenosine; APC, adenosine-enhanced ischemic preconditioning; CCPA, 2-chloro-N(6)-cyclopentyladenosine; SAP, systemic arterial pressure; LVSP, left ventricular systolic pressure; HR, heart rate; CSF, coronary sinus flow; CK, creatine kinase; LDH, lactate dehydrogenase; ET, endothelin; MDA, malondialdehyde; SOD, superoxide dismutase; ATP, adenosine triphosphate; IR, ischemia/reperfusion; +dp/dt, increase in pressure over time; -dp/dt, decrease in pressure over time.

**Table IV.** Relative mRNA expression of Bcl-2 and ICAM-1.

Groups	Bcl-2/GADPH	ICAM-1/GADPH
ST	1.00	1.00
ADO + ST	1.01±0.01	1.03±0.04
APC + ST	1.42±0.07^a^	0.70±0.03^a^
CCPA + ST	1.16±0.02^a,b^	0.85±0.02^a,b^
CCPA + ADO + ST	1.40±0.05^a,c^	0.72±0.05^a,c^
CCPA + APC + ST	2.59±0.07^a–d^	0.47±0.07^a–d^

Data are presented as the mean ± standard error.

a P<0.05 vs. the ST group

b P<0.05 vs. the APC + ST group

c P<0.05 vs. the CCPA + ST group

d P<0.05 vs. the CCPA + ADO + ST group. Bcl-2, B-cell lymphoma-2; ICAM-l, intercellular adhesion molecule-1; ST, St. Thomas II; ADO, adenosine; APC, adenosine-enhanced preconditioning; CCPA, 2-chloro-N(6)-cyclopentyladenosine.
